# Segmental inner macular layer analysis with spectral-domain optical coherence tomography for early detection of normal tension glaucoma

**DOI:** 10.1371/journal.pone.0210215

**Published:** 2019-01-10

**Authors:** Jih-Pin Lin, Pei-Wen Lin, Ing-Chou Lai, Jen-Chia Tsai

**Affiliations:** 1 Department of Ophthalmology, Kaohsiung Chang Gung Memorial Hospital, Kaohsiung, Taiwan, R.O.C; 2 Chang Gung University College of Medicine, Kaohsiung, Taiwan, R.O.C; Singapore National Eye Centre, SINGAPORE

## Abstract

**Purpose:**

To segment the inner macular layers (IML) and compare the discriminating abilities of the macular and peripapillary retinal nerve fiber layer (mRNFL and pRNFL, respectively) thicknesses in patients with early-stage normal tension glaucoma (NTG).

**Design:**

Cross-sectional study

**Methods:**

Forty-nine normal subjects and 69 preperimetric glaucoma (PPG) and 60 NTG patients were enrolled. Spectral-domain optical coherence tomography (SD-OCT) was used to obtain pRNFL and macular thickness parameters and segment the IML in all subjects. Area under the receiver operating characteristic (AUROC) curves were used to compare the diagnostic capabilities of different parameters.

**Results:**

The pRNFL, total macular layer (TML), mRNFL, and macular ganglion cell layer (mGCL) were significantly thinner in the NTG group than in the PPG and normal groups. The global and superotemporal pRNFL and the mGCL in the superior outer area were the three best parameters for detecting early NTG. The discriminating capabilities of the superior and inferior mGCL were comparable to those of the corresponding pRNFL (*p* = 0.573, 0.841). Concerning location, the mGCL had higher AUROCs in the outer sectors (0.863, 0.837) than in the inner sectors (0.747, 0.747). Pearson’s correlation coefficients also revealed significant correlations between the mGCL and pRNFL (superior: r = 0.499, inferior: r = 0.624). The strongest correlation was between the mGCL and mean deviation (MD) (superior: r = 0.434 and inferior: r = 0.402).

**Conclusions:**

The diagnostic value of mGCL thickness is comparable to that of pRNFL thickness. IMLs in the outer sectors had better diagnostic capabilities than those in the inner sector for detecting early NTG.

## Introduction

Glaucoma is the leading cause of irreversible blindness worldwide. Glaucoma is associated with the progressive loss of retinal ganglion cells (RGC), thinning of the retinal nerve fiber layer (RNFL), notching of the optic nerve head (ONH) and characteristic visual field (VF) defects. Some studies [1–3] have reported that the pathological structural damage observed in glaucoma can be detected several years before visual field defects occur. Previous studies [4,5] have revealed that axonal degeneration may precede RGC body death. However, oxidative stress can lead to cell body death in the retina independent of axonal degeneration [4,5]. The cell bodies are located in the ganglion cell layer (GCL), and their axons are situated in the RNFL [6]. Early thinning of the macular ganglion cell-inner plexiform layer (mGCIPL) or macular ganglion cell complex (mGCC) has been noted in glaucoma patients [1,6–8]. Although many studies have explored the use of diagnostic techniques in the macula, few reports have performed layer-by-layer segmented mGCL analyses.

A collaborative normal tension glaucoma study group established that intraocular pressure (IOP) is part of the pathogenic process underlying normal tension glaucoma (NTG) [9] and that some pressure-independent vascular factors, such as vascular dysregulation and ischemia, are more important during the development and progression of NTG than primary open-angle glaucoma (POAG) [10]. Several reports have shown that there are structural and functional differences between NTG and POAG [11–13]. For example, a deeper and more central VF defect closer to the fixation point is more commonly observed in NTG than in POAG. In addition, nearly half of RGCs are located within the macula [14,15], and the macula is generally less variable than the ONH and peripapillary retinal nerve fiber layer (pRNFL). Since NTG can be associated with involvement of the central VF, significant macular RGC loss may be detected during the early stage of NTG. Few years ago, in the early stage of studying macula analysis, NTG and POAG were not separate clearly into two study groups, and few studies focus in NTG patients.

Optical coherence topography (OCT) was first introduced in 1991 and has been widely used to detect structural changes since 2002. The advent of spectral-domain OCT (SD-OCT) allowed a higher scan resolution and faster speed than could be achieved previously using OCT and enabled better quantitative mGCC assessment, allowing for the effective diagnosis and evaluation of glaucoma progression. Spectralis OCT (Heidelberg Engineering, GmbH) provides an analysis that can automatically segment the TML into ten retinal layers. Monitoring the thickness of an isolated macular layer may improve the early detection of glaucoma in a clinical setting.

We used Spectralis OCT new segmentation software to automatically segment the inner macular layers (IMLs) and to subsequently compare the ability to discriminate glaucoma by analyzing macular and pRNFL thickness parameters in patients with early stage NTG.

## Methods

In this cross-sectional study, we investigated 49 normal subjects, 69 preperimetric glaucoma subjects (PPG) and 65 patients with early NTG. All included individuals were regularly followed-up at the Glaucoma Clinic at Kaohsiung Chang Gung Memorial Hospital. Informed consent in written form was obtained from all of the subjects and signed after our explanation. The design of this study adhered to the tenets of the Declaration of Helsinki and was reviewed and approved by the institutional review board and ethics committee of Chang Gung Memorial Hospital.

All subjects underwent a thorough ophthalmologic examination, including best-corrected visual acuity (BCVA), refraction, IOP measurement with Goldmann applanation tonometry, slit-lamp biomicroscopy, gonioscopy, central corneal thickness (CCT), ophthalmoscopy, red-free fundus photography (TRC-50EX, TOPCON, Japan), standard automatic perimetry (SAP) and SD-OCT exam. The refraction was expressed as spherical equivalence (SE), which was calculated as a sphere plus half of the cylinder. CCT was measured on a Non-Contact Specular Microscope (SP-3000P, TOPCON, Tokyo, Japan), and SAP examinations were performed with a Swedish Interactive Threshold Algorithm standard 30–2 Humphrey field analyzer (HFA, Carl Zeiss Meditec, Dublin, CA). Unreliable VF tests with a fixation loss of more than 20% and a false-positive or false-negative rate of more than 15% were excluded. A glaucomatous VF defect was defined according to Hodapp-Parrish-Anderson criteria. Glaucomatous VF defects were confirmed by two reliable VF exams. A glaucomatous optic disc was defined by the presence of thinning or notching in the neuroretinal rim, excavation of the optic disc or the presence of disc hemorrhage on stereoscopic color fundus photographs.

The inclusion criteria were as follows: patients had a BCVA of 20/40 or better; spherical refraction within ±6.0 diopters; cylinder correction within ±3.0 diopters; open angle on gonioscopy; and an IOP less than 21 mmHg. Early stage was defined as a mean deviation (MD) value greater than -6 dB on the SAP exam. Exclusion criteria were patients who had corneal lesions, chronic uveitis, secondary glaucoma, optic neuropathy other than glaucoma, retinal pathology, maculopathy and previous ocular trauma history. Patients with low reliability of the VF test results, OCT image quality less than 20 or insufficient ophthalmic information were also excluded.

NTG was defined as an IOP less than 21 mmHg on more than two occasions without medication, a glaucomatous optic disc, RNFL defects with corresponding glaucomatous VF defects and open anterior chamber angles on gonioscopy. Normal participants (N) aged between 20 and 60 years old were recruited from hospital staff or among patients who came for a routine eye examination and had an IOP <21 mmHg, a normal-appearing optic disc and an absence of VF defects. PPG was defined as having an IOP <21 mmHg, an open angle on gonioscopy, and large disc cupping with a cup/disc ratio >0.6 but without corresponding glaucomatous VF defects.

10.6084/m9.figshare.7497128

https://figshare.com/s/f0e00d5e7f73b9a93bea

All files are available from the figshare database.

### Spectral domain optical coherence tomography

SD-OCT imaging was performed with a Spectralis OCT (Heidelberg Engineering, GmbH). The acquisition rate of the Spectralis OCT is 40,000 A scans per second. The optical depth resolution is 7 μm, and the digital transverse and axial resolutions are 14 and 3.9 μm, respectively. The scan circle is 3.6 mm in diameter. The pRNFL values were divided into 4 quadrants, and the superior and inferior quadrants were further divided into nasal and temporal sectors. Each patient underwent scans to measure pRNFL and macular thickness during the same visit. The OCT parameters, including global and regional pRNFL thickness, were generated in the analysis reports.

**Fig 1:**
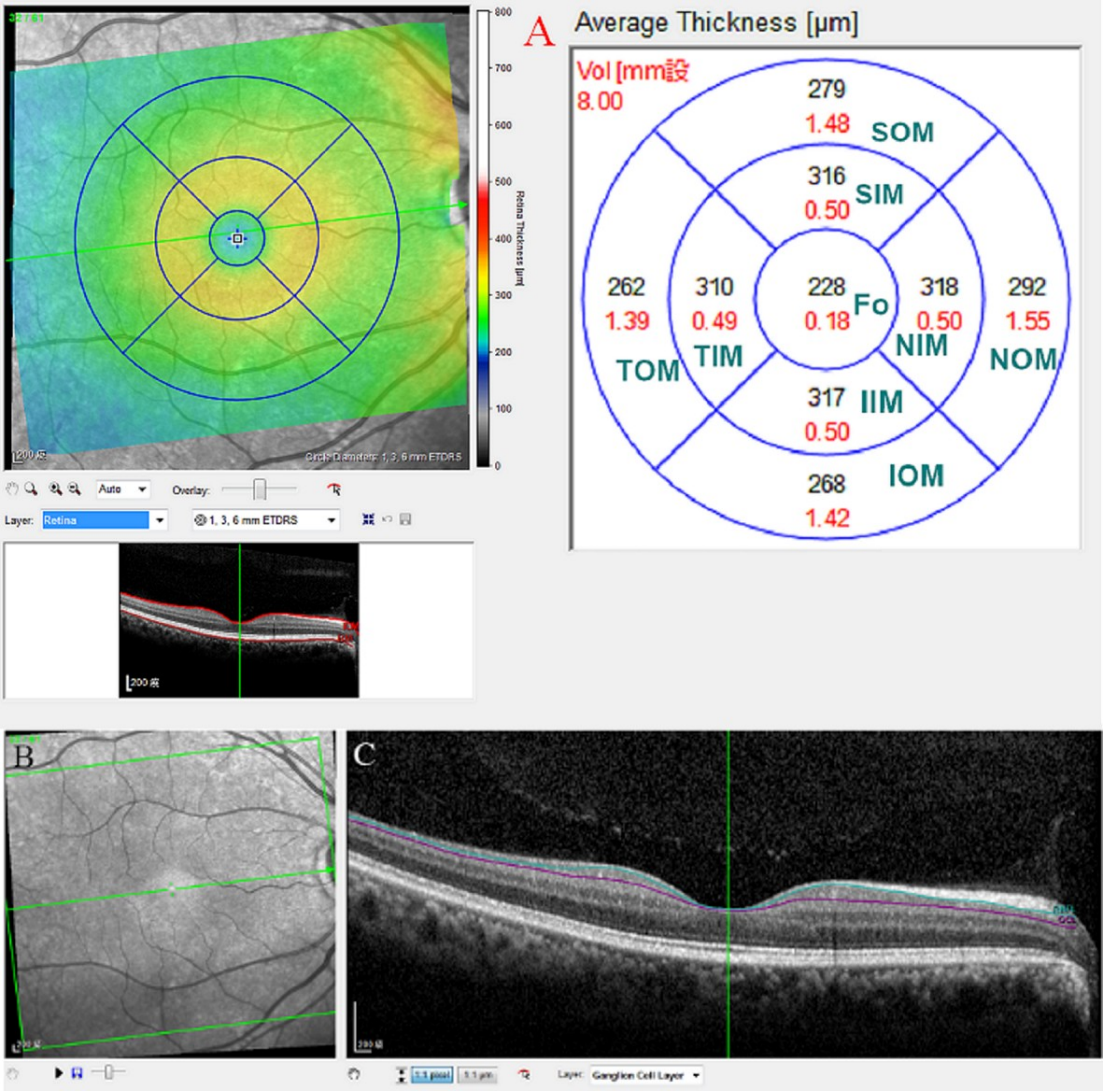
Layer-by-layer segmentation was executed automatically using the new software for the Spectralis OCT. (A) Nine macular sectors of every single retinal layer in spectral-domain optical coherence tomography. The superior outer macula, superior inner macula, fovea, inferior inner macula and inferior outer macula were used for analysis. (B) Infrared reflectance image of the macular region. (C) Segmented macular ganglion cell layers in B scan images. ** SIM: superior inner macula, IIM: inferior inner macula, TIM: temporal inner macula, NIM: nasal inner macula; SOM: superior outer macula, IOM: inferior outer macula, TOM: temporal outer macula, NOM: nasal outer macula, fo: fovea, mRNFL: macular retinal nerve fiber layer, mGCL: macular ganglion cell layer.

Using a specific protocol (Heidelberg Eye Explorer version 1.8.6.0, Spectralis Viewing Module 6.3.4.0; Heidelberg Engineering, GmbH), the average retinal thickness within a 1-mm radius of the central fovea was obtained, calculated based on an Early Treatment Diabetic Retinopathy Study grid. Two concentric circles with diameters of 3 mm and 6 mm were drawn outside the central circle of the fovea and used to represent the inner and outer macular areas, respectively (**Fig 1A**). The concentric circles were further divided into superior, temporal, inferior, and nasal quadrants. Five sectorial retinal thickness measurements were acquired by OCT at the fovea and superior outer, superior inner, inferior outer and inferior inner regions for further analysis. After the TML thickness was measured, segmentation of the TML was automatically performed by the new segmentation tools that were provided by the manufacturer of the Spectralis OCT (**Fig 1B and 1C**). Because glaucoma mainly affects the IMLs, we chose to analyze the macular RNFL (mRNFL) and macular GCL (mGCL). To avoid errors in interpretation, no manual correction was applied to the OCT output. Instead, images with an OCT image quality <20 (n = 37) or images in which the delineation was questioned (n = 22) were excluded from this study before the analysis was performed.

All examinations were completed within a 6-month period in each patient. If both eyes fulfilled the inclusion criteria, the eye with the best OCT image quality was used for the analysis. We chose parameters at the superior and inferior quadrants for the statistical analysis, as they are commonly affected in glaucoma. The superior macular thickness was defined as the average thickness of the superior-outer macular (SOM) and superior-inner macular (SIM) sectors. The inferior macular thickness was defined as the average thickness of the inferior-outer macular (IOM) and inferior-inner macular (IIM) sectors.

### Statistical analysis

The characteristics of the participants were assessed by one-way analysis of variance (ANOVA), and the χ^2^ test was used to analyze the gender parameter. A post hoc test with Scheffe adjustment was used to determine significance between any two groups. One-way ANOVA was also conducted to assess differences in the thicknesses of the pRNFL, TML, mRNFL, and mGCL among the groups. The normal distribution was verified using a histogram test. Statistical associations among macular values, pRNFL, and visual function were evaluated by Pearson’s correlation coefficient. Areas under the receiver operating characteristic (AUROC) curves were used to assess the diagnostic capabilities of retinal layers. Significant differences between AUROCs were calculated using the DeLong test. A *p* value <0.05 represents a significant difference. All statistical analyses were performed in SPSS statistical software (version 18.0; SPSS, Inc., Chicago, IL), except the DeLong test, which was performed in MedCalc-statistical-software (Version 16.8.4).

## Results

This study included 49 healthy subjects, 69 subjects with PPG and 60 patients with NTG. The demographic data is shown in **Table 1**. After Scheffe correction, there were no significant differences in age or gender between the N group and the PPG, CCT, and SE groups or in IOP between the N group and the PPG group. However, IOP was higher in the N group than in the NTG group, potentially because IOP-lowering agents were used in NTG patients. As expected, MD and PSD were significantly worse in the NTG group than in the PPG and N groups, and this relationship exhibited a linear trend.

**Table 1:** Demographic data of normal subjects, preperimetric glaucoma subjects and normal tension glaucoma patients.

	Normal (n = 49)	PPG (n = 69)	NTG (n = 60)	p*	Multiple comparison^†^
**Age (years)**	51.73±11.26	53.71±15.09	57.58±12.90	0.065	
**Sex^‡^**				0.001	
**Male**	12(24.5%)	23(33.3%)	35(58.3%)		NTG>PPG = N (male)
**Female**	37(75.5%)	46(66.7%)	25(41.7%)		
**CCT (μm)**	543.63±32.76	540.85±48.84	531.45±40.84	0.312	
**SE (diopters)**	-1.72±2.39	-1.70±2.46	-1.70±2.63	0.999	
**IOP (mmHg)**	15.08±3.68	14.22±3.35	12.85±3.16	0.003	N>NTG
**VF**					
**MD (dB)**	-0.42±0.93	-1.18±1.42	-2.98±1.59	<0.001	N>PPG>NTG ^a^
**PSD (dB)**	1.88±0.52	2.17±1.04	3.53±1.83	<0.001	NTG>PPG>N^b^

p*: *p* value among three groups (assessed by one-way analysis of variance)

† Value for comparison of normal tension glaucoma and preperimetric glaucoma subjects, normal tension glaucoma and normal subjects, normal subjects and preperimetric glaucoma subjects (multiple comparisons with Scheffe correction).

‡ χ^2^ test.

a: linear trend (assessed by one-way analysis of variance).

b: linear trend (assessed by one-way analysis of variance), *p* value for comparison of N and PPG: 0.482.

CCT: central corneal thickness; SE: spherical equivalence; IOP: intraocular pressure; VF: visual field; MD: mean deviation; PSD: pattern standard deviation; N: normal subjects; PPG: preperimetric glaucoma subjects; NTG: normal tension glaucoma.

The thickness parameters among the three groups in the pRNFL, TML, mRNFL and mGCL groups are shown in **Table 2**. There were significant differences in pRNFL thickness parameters between the N and NTG groups and between the PPG and NTG groups. Regarding TML parameters, the TML was significantly thinner in both the SOM and IOM layers in the PPG group than in the N group. Regarding mRNFL parameters, there were significant differences in the thicknesses of the SOM and IOM layers between the N and NTG groups and between the PPG and NTG groups. A comparison of mGCL parameters revealed that there were significant differences in the thicknesses of the SOM, IOM, SIM, and IIM between the N and NTG groups and between the PPG and NTG groups.

**Table 2:** pRNFL, TML, mRNFL and mGCL thicknesses in normal subjects, preperimetric glaucoma subjects and normal tension glaucoma patients.

Thickness (um)	Normal (n = 49)	PPG (n = 69)	NTG (n = 60)	p* value	N. vs. PPG (*p*)	N. vs NTG (*p*)	PPG vs NTG (*p*)
**pRNFL**							
**Global**	105.94±11.27	97.32±10.08	83.40±14.74	**<0.001**	**0.001**	**<0.001**	**<0.001**
**Superior**	132.55±20.80	122.15±16.53	102.63±22.92	**<0.001**	**0.023**	**<0.001**	**<0.001**
**Inferior**	131.61±16.27	125.38±16.35	102.90±25.55	**<0.001**	0.248	**<0.001**	**<0.001**
**Temp-sup**	152.39±19.62	140.55±17.13	114.37±26.94	**<0.001**	**0.015**	**<0.001**	**<0.001**
**Temp-inf**	162.02±20.21	149.32±19.46	118.10±35.65	**<0.001**	**0.037**	**<0.001**	**<0.001**
**Nas-sup**	112.33±25.89	103.32±20.67	90.45±25.98	**<0.001**	0.137	**<0.001**	**0.011**
**Nas-inf**	100.73±21.08	101.00±21.57	87.33±21.82	**0.001**	0.998	**0.006**	**0.002**
**TML**							
**SOM**	301.84±14.21	292.12±12.70	285.15±23.57	**<0.001**	**0.013**	**<0.001**	0.081
**SIM**	339.82±15.39	333.67±15.22	328.58±19.27	**0.003**	0.148	**0.003**	0.231
**Fovea**	255.59±18.40	263.73±22.78	262.92±23.39	0.108	0.141	0.224	0.978
**IIM**	335.12±14.65	329.96±13.71	323.30±20.78	**0.001**	0.255	**0.001**	0.080
**IOM**	287.80±15.07	279.12±12.65	267.60±20.83	**<0.001**	**0.020**	**<0.001**	**0.001**
**mRNFL**							
**SOM**	41.84±5.54	38.12±5.22	34.23±6.50	**<0.001**	**0.003**	**<0.001**	**0.001**
**SIM**	24.69±3.45	23.48±3.30	22.82±3.31	**0.015**	0.154	**0.016**	0.535
**Fovea**	10.24±1.97	10.68±2.05	11.15±2.15	0.076	0.529	0.078	0.439
**IIM**	25.53±3.73	24.67±2.94	23.73±3.64	**0.025**	0.401	**0.026**	0.303
**IOM**	43.04±5.19	39.99±5.53	32.97±8.34	**<0.001**	**0.046**	**<0.001**	**<0.001**
**mGCL**							
**SOM**	37.18±2.99	34.41±2.48	32.37±3.26	**<0.001**	**<0.001**	**<0.001**	**0.001**
**SIM**	52.90±3.78	50.87±4.39	48.00±6.48	**<0.001**	0.103	**<0.001**	**0.007**
**Fovea**	12.39±3.05	13.43±3.18	13.65±4.04	0.136	0.272	0.169	0.940
**IIM**	52.16±3.36	50.54±3.71	46.02±8.40	**<0.001**	0.311	**<0.001**	**<0.001**
**IOM**	34.12±3.50	31.97±2.94	28.87±4.14	**<0.001**	**0.006**	**<0.001**	**<0.001**

* pRNFL: peripapillary retinal nerve fiber layer; TML: total macular layer; mRNFL: macular retinal nerve fiber layer; mGCL: macular ganglion cell layer; N: normal subjects; PPG: preperimetric glaucoma subjects; NTG: normal tension glaucoma; Temp-sup: temporal-superior; Temp-inf: temporal-inferior; Nas-sup: nasal-superior; Nas-inf: nasal-inferior; SIM: superior inner macula; IIM: inferior inner macula; SOM: superior outer macula; IOM: inferior outer macula.

Pearson’s correlations between the MD in VF and the differences in retinal thickness are shown in **Table 3**. There were moderate correlations between the mGCL and MD in the superior and inferior quadrants (r = 0.434, r = 0.402, respectively), which were the strongest correlations among all of the parameters. We also compared the correlations between the mGCL and pRNFL in the superior (r = 0.499) and inferior (r = 0.624) quadrants, as shown in **Table 3**.

**Table 3:** Correlations of pRNFL, TML, mRNFL and mGCL thickness with MD in superior and inferior sectors.

Variables		Correlation with MD (r)	Correlation with pRNFL (r)
**pRNFL**	**Superior**	0.340*	NA
	**Inferior**	0.371*	NA
**TML**	**Superior**	0.315*	0.302*
	**Inferior**	0.345*	0.499*
**mRNFL**	**Superior**	0.323**	0.251*
	**Inferior**	0.359*	0.463*
**mGCL**	**Superior**	0.434*	0.423*
	**Inferior**	0.402*	0.624*

*p<0.001

**p = 0.001

AUROCs with 95% confidence intervals and sensitivity values are shown for the pRNFL, TML, mRNFL and mGCL thickness parameters that differentiated NTG eyes from N eyes in **Table 4**. The global pRNFL had the highest AUROCs (0.896). With regard for the macular parameters, the highest AUROC among IMLs was the mGCL in the SOM (AUROC = 0.863), and the diagnostic impacts of the superior and inferior mGCL were similar to that of the pRNFL (*p* = 0.573 and 0.841, respectively). AUROCs with 95% confidence intervals and sensitivity values are shown for the pRNFL, TML, mRNFL and mGCL thickness parameters that differentiated PPG eyes from N eyes in **Table 5**. The mGCL in the SOM had the highest AUROCs (0.759), followed by global pRNFL (0.726) and superior mGCL (0.715). In the present study, the mGCL was the IML parameter that was the most similar to pRNFL in diagnostic accuracy.

**Table 4:** Diagnostic capabilities of pRNFL, TM, mRNFL and mGCL for differentiating early normal tension glaucoma.

Variables (μm)	Sensitivity at 80% Specificity (%)	Sensitivity at 95% Specificity (%)	AUROC (95%CI)95% CI	*p* value
**pRNFL thickness**				
**Global**	80.0	38.2	**0.896(0.838–0.954)**	<0.001
**Superior**	77.3	58.3	0.859(0.790–0.928)	<0.001
**Inferior**	69.8	50.8	0.828(0.753–0.904)	<0.001
**Temp-sup**	80.0	64.8	**0.882(0.820–0.945)**	<0.001
**Temp-inf**	76.5	55.0	0.858(0.789–0.927)	<0.001
**Nas-sup**	58.0	32.1	0.742(0.650–0.834)	<0.001
**Nas-inf**	33.3	22.4	0.646(0.543–0.749)	0.009
**TML thickness**				
**SOM**	72.6	38.2	**0.820(0.741–0.899)**	<0.001
**SIM**	44.0	22.4	0.673(0.572–0.774)	0.002
**Fovea**	16.4	3.3	0.421(0.314–0.528)	0.157
**IIM**	44.8	22.9	0.671(0.570–0.771)	0.002
**IOM**	70.3	48.7	0.794(0.710–0.877)	<0.001
**Superior**	58.3	30.0	0.760(0.670–0.850)	<0.001
**Inferior**	60.7	33.3	0.753(0.663–0.844)	<0.001
**mRNFL thickness**				
**SOM**	64.2	49.8	0.810(0.731–0.889)	<0.001
**SIM**	34.6	23.0	0.639(0.536–0.742)	0.006
**Fovea**	12.4	3.8	0.373(0.268–0.479)	0.031
**IIM**	33.0	16.8	0.621(0.517–0.726)	0.028
**IOM**	68.5	53.3	**0.840(0.769–0.912)**	<0.001
**Superior**	63.3	42.1	0.782(0.698–0.867)	<0.001
**Inferior**	59.8	43.0	0.796(0.715–0.877)	<0.001
**mGCL thickness**				
**SOM**	78.0	49.5	**0.863(0.794–0.931)**	<0.001
**SIM**	59.6	27.4	0.747(0.655–0.839)	<0.001
**Fovea**	11.8	3.3	0.424(0.317–0.532)	0.176
**IIM**	55.2	44.8	0.747(0.656–0.838)	<0.001
**IOM**	70.0	48.9	0.837(0.764–0.911)	<0.001
**superior**	67.6	46.3	0.834(0.760–0.909)	<0.001
**Inferior**	74.0	48.0	0.836(0.761–0.912)	<0.001

pRNFL: peripapillary retinal nerve fiber layer; TML: total macular layer; mRNFL: macular retinal nerve fiber layer; mGCL: macular ganglion cell layer; Temp-sup: temporal-superior; Temp-inf: temporal-inferior; Nas-sup: nasal-superior; Nas-inf: nasal-inferior; SIM: superior inner macula; IIM: inferior inner macula; SOM: superior outer macula; IOM: inferior outer macula; AUROC: area under the receiver operating characteristic; CI: confidence interval.

**Table 5:** Diagnostic capabilities of pRNFL, TM, mRNFL and mGCL for differentiating preperimetric glaucoma.

Variables (μm)	Sensitivity at 80% Specificity (%)	AUROC (95% CI)	*p* value
**pRNFL thickness**			
**Global**	58.5	**0.726 (0.636–0.804)**	<0.001
**Superior**	37.4	0.647 (0.554–0.733)	0.004
**Inferior**	33.3	0.616 (0.522–0.704)	<0.026
**Temp-sup**	44.8	0.687 (0.595–0.769)	<0.001
**Temp-inf**	56.4	0.695 (0.604–0.777)	<0.001
**Nas-sup**	32.5	0.579 (0.485–0.670)	0.139
**Nas-inf**	15.7	0.507 (0.413–0.600)	0.900
**TML thickness**			
**SOM**	42.9	0.696 (0.605–0.778)	<0.001
**SIM**	33.0	0.633 (0.540–0.720)	0.011
**Fovea**	29.3	0.590 (0.496–0.680)	0.089
**IIM**	27.5	0.615 (0.521–0.703)	0.029
**IOM**	44.1	0.663 (0.570–0.748)	0.002
**Superior**	38.8	0.657 (0.564–0.742)	0.002
**Inferior**	34.2	0.647 (0.554–0.733)	0.005
**mRNFL thickness**			
**SOM**	49.0	0.693 (0.601–0.775)	<0.001
**SIM**	43.5	0.597 (0.503–0.687)	0.060
**Fovea**	37.8	0.562 (0.468–0.653)	0.245
**IIM**	22.7	0.565 (0.470–0.656)	0.237
**IOM**	34.9	0.643 (0.549–0.729)	0.006
**Superior**	44.5	0.668 (0.575–0.752)	0.001
**Inferior**	33.0	0.639 (0.545–0.725)	0.001
**mGCL thickness**			
**SOM**	59.5	**0.759 (0.671–0.833)**	<0.001
**SIM**	44.2	0.648 (0.555–0.734)	0.004
**Fovea**	36.6	0.589 (0.494–0.678)	0.097
**IIM**	39.6	0.641 (0.548–0.727)	0.006
**IOM**	39.1	0.681 (0.589–0.764)	<0.001
**superior**	50.1	**0.715 (0.625–0.794)**	<0.001
**Inferior**	48.9	0.699 (0.607–0.780)	<0.001

pRNFL: peripapillary retinal nerve fiber layer; TML: total macular layer; mRNFL: macular retinal nerve fiber layer; mGCL: macular ganglion cell layer; Temp-sup: temporal-superior; Temp-inf: temporal-inferior; Nas-sup: nasal-superior; Nas-inf: nasal-inferior; SIM: superior inner macula; IIM: inferior inner macula; SOM: superior outer macula; IOM: inferior outer macula; AUROC: area under the receiver operating characteristic; CI: confidence interval.

**Fig 2:**
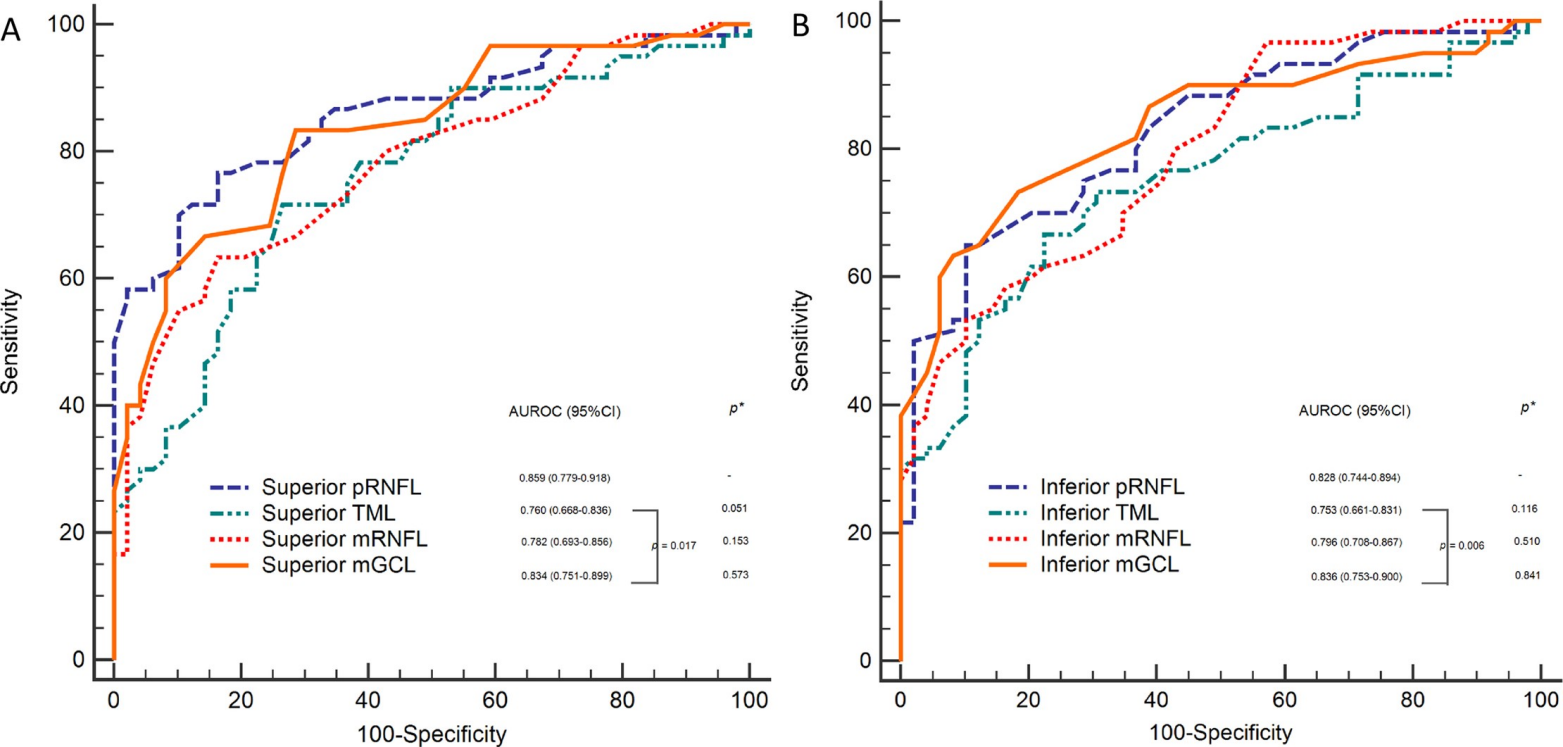
Areas under the receiver operating characteristic curves of the peripapillary retinal nerve fiber layer, total macular layer, macular retinal nerve fiber layer and macular ganglion cell layer in the superior A and inferior B quadrants, respectively. *p: The discriminating abilities of the superior and inferior inner macular layers vs. Prnfl. **pRNFL: peripapillary retinal nerve fiber layer, TML: total macula layer, mRNFL: macular retinal nerve fiber layer, mGCL: macular ganglion cell layer, AUROC: area under the receiver operating characteristic.

A comparison of AUROCs between the pRNFL and the macular layers was performed with the DeLong test, and the results are shown in **Fig 2**. In early NTG, there were no differences between the pRNFL and IML parameters (**Fig 2**). However, differences were detected between the TML and mGCL in the corresponding sectors (*p* = 0.017, 0.006; **Fig 2**). The discriminating abilities of the superior and inferior mGCL were comparable to those of the corresponding pRNFL (*p* = 0.573, 0.841; **Fig 2**).

## Discussion

Kim [16] showed that it may be more practical and significant to measure IMLs than to measure pRNFL when seeking a glaucoma diagnosis because the IMLs are involved at an earlier stage. Moreover, the level of variation in mGCL thickness measurements was lower than that of conventional pRNFL and optic disc parameters since the disc tilting and torsion are common. We used Spectralis OCT to acquire an image of a single IML, and the data in this image was compared to data for the pRNFL among the N, PPG and NTG groups. We aimed to determine whether this approach is a useful clinical tool that can help us to improve the NTG diagnosis rate in the early stage (because of the high prevalence of NTG in our country). This is one of only a few studies to include PPG patients in the evaluation of the utility of measuring a single IML to diagnose NTG.

### Retinal thickness

Pazos’s [17] found that the TML and mRNFL were thinner mainly in their outer sectors, similar to our results. Tan [18] found that in both glaucoma and PPG patients, the reduction observed in the thickness of the IMLs was more severe than the reduction observed in TML thickness. These data indicate that the mRNFL and mGCL are the primary sites affected in glaucoma. Nakano [19] showed that the mean thickness of the mGCL was significantly thinner than those of the mRNFL and pRNFL, further indicating that the thickness of the mGCL is more sensitive than that of the pRNFL.

With regard for location, we found that the thickness changed more in the outer than in the inner sector, consistent with Pazos [17]. The pathological explanation for this result is unclear. To determine which layer was most affected by glaucoma, we used fractional deviations [18] to identify which regions are more vulnerable in glaucoma ([average IML thickness of N eyes—the average IML thickness of NTG eyes]/ average IML thickness of N eyes). The fractional deviations of the mGCL in the SOM, IOM, SIM and IIM were 12.9%, 15.4%, 9.3% and 11.8%, respectively. The fractional deviations of the mRNFL in the SOM, IOM, SIM and IIM were 18.2%, 23.4%, 7.6% and 7.1%, respectively. Accordingly, the outer IMLs were more affected than the inner sectors in the same macular layer. The more affected outer macular sectors were the same locations in which early thinning of the inferior and superior neuroretinal rim of the disc were observed. The collection of vulnerable superior and inferior retinal arcuate axons that travel through the temporal hemimacula to the superior or inferior aspect of the disc results in the thinning observed in the neuroretinal rim of the disc. Further longitudinal studies are required to investigate the related pathological changes in the outer and inner portions.

### Diagnostic performance

Firat [20] found that all mGCC parameters demonstrated diagnostic capabilities similar to those of pRNFL when used to discriminate NTG. Kim [21] analyzed mGCC and pRNFL parameters in moderate-stage NTG patients and found that the inferior mGCC and inferior pRNFL were the two best parameters for discriminating NTG (AUROC = 0.875 and 0.846). Nakano [19] found that in PPG, the sensitivity of the mGCL was even higher than pRNFL thickness when assessed via Spectralis OCT (83.8% vs. 54.1%). Recently, Edlinger [22] found that both mRNFL and pRNFL produced equal diagnostic performance in a high-tension perimetric glaucoma group (88.5 and 96.2%). However, in the NTG group, the mRNFL was inferior to all other layers, similar to our results.

In contrast to our findings, previous studies [7,23] showed that the diagnostic value of mRNFL is higher than that of either pRNFL or mGCL. This conclusion was based on data obtained using prototype software [7,23] that had a lower segmentation accuracy than the most recent version, which was used in this study. Two systemic reviews [24,25] concluded that pRNFL remained preferable to macular parameters for diagnosing glaucoma. However, at that time, it was not possible to segment the mGCC into a single layer; therefore, these two reviews were not able to compare the diagnostic power of the pRNFL and mGCL. The differences in diagnostic performance between high-tension glaucoma and NTG were also assessed in recent years. Edlinger’s [22] results showed that mRNFL had high diagnostic value in the inferior sectors, especially in high tension glaucoma groups, but was outperformed by the other layers in the NTG groups, which were not evaluated by the two systemic reviews [24,25]. Recently, Kim [26] used a similar study design and new segmentation software and found that the best parameter was the inferotemporal (IT) mGCL (0.938). The AUROCs were higher for the global mRNFL and the global mGCL (0.915 and 0.914, respectively) than for the pRNFL (0.878). Pazos [17] used the same software with the latest version and found that pRNFL (0.956) still performed better than other macular parameters. The isolated mGCL had less diagnostic capability (0.858; *p*<0.005). Pazos [17] suggested that the differences between their results and those presented in Kim [26] were caused by differences in the populations and disease severities that were studied and because global pRNFL is not equal to regional macular parameters, which underestimate the diagnostic power of pRNFL. We used the same OCT and a similar version of the segmentation software, evaluated a similar disease severity (-2.98±1.59 dB vs. -2.26±1.82 dB) to Pazos [17] and explored the more equal comparison between the pRNFL and mGCL. We found that the mGCL has a diagnostic capacity similar to that of pRNFL, in accordance with Edlinger’s [22] and Kim’s [26] results. The only difference between ours, Kim’s [26] and Pazos [17] was in the study populations. The population evaluated in Pazos [17] study was all white. Caucasians have been shown to have a higher prevalence of POAG. Edlinger’s [22] study did take different types glaucoma into consideration, and the results associated with normotensive perimetric glaucoma obtained in their study was similar to that obtained in our study (diagnostic performance: mGCL = pRNFL>mRNFL) but different from our result for high-tension glaucoma (diagnostic performance: pRNFL = mGCL = mRNFL). Different types of glaucoma and ethnic variety make it difficult to compare diagnostic power between the pRNFL and macular parameters among previous studies.

### Structure-function relationships

Moreover, significant structure-function relationships were found in eyes exhibiting glaucomatous damage. Recently, a similar study performed using the same new segmentation software [17] found that in POAG, the parameters pRNFL, TML, mRNFL, and mGCL were significantly correlated with MD (mGCL>pRNFL>TML>mRNFL). Another similar cross-sectional study [27] also used Spectralis OCT and found that GCL thickness showed the strongest structure-function correlation in early glaucoma, while IPL thickness showed the strongest structure-function correlation in moderate to advanced glaucoma. Our findings are therefore similar to those reported in the previous literature [17,27].

### Correlation between pRNFL and IMLs

Additionally, the structural changes observed in the thickness of the mGCL were significantly correlated with pRNFL in early NTG, especially in the inferior sectors (r = 0.624). To date, no reports have used Spectralis OCT to explore the correlation between a single IML and the pRNFL in early NTG. Although a direct comparison with other studies is problematic because different devices and study designs were used, we found that the correlation between IML and pRNFL thickness was strong, especially in the inferior sector. The strong correlation found in this study may be mainly attributable to the correlation between the thicknesses of specific layers of the macula and that of the pRNFL rather than that of TML. Among the IMLs, the mGCL was much more strongly correlated with pRNFL than with mRNFL and TML.

There were some limitations to our study. First, we used auto-segmentation results without manual corrections. Considering the small percentage of automated errors [17], we chose high-quality OCT images in which the quality score was above 20, and images whose quality was questioned were excluded from this study. Second, we included only Taiwanese patients, and the results of these analyses might vary among different populations. Third, our study was a cross-sectional evaluation of patients with early glaucoma. Patients with moderate and severe glaucoma may present different changes in the mGCL and mRNFL. Further studies that include patients with different severities of glaucoma are needed to identify the changes that occur in the IML in a broader range of patients. Finally, this study was limited by its small sample size. Moreover, participants were enrolled from a tertiary center, and these results may therefore not be representative of the whole population. Further studies that include larger sample sizes should be carried out to provide more statistical certainty.

In conclusion, the diagnostic ability of mGCL thickness is comparable to pRNFL thickness in Taiwanese with early NTG. The diagnostic capacities of the outer sectors of the IMLs were better than those of the inner sectors for detecting early glaucoma. Measurements of segmented mGCL may be an alternative or supplemental tool for improving the early detection of NTG.

## Abbreviations

ANOVA: one way analysis of variance
AUROC: area under receiver operating characteristic curve
CCT: central corneal thickness
IIM: inferior-inner macular sectors
IML: inner macular layers
IOM: inferior-outer macular sectors
IOP: intraocular pressure
IPL: inner plexiform layer
IT: inferotemporal
MD: mean deviation
mGCC: macular ganglion cell complex
mGCL: macular ganglion cell layer
mGCIPL: macular ganglion cell-inner plexiform layer
mRNFL: macular retinal nerve fiber layer
NTG: normal tension glaucoma
OCT: optical coherence tomography
ONH: optic nerve head
POAG: primary open-angle glaucoma
PPG: preperipapillary glaucoma
pRNFL: peripapillary retinal nerve fiber layer
RGC: retinal ganglion cells
SAP: standard automatic perimetry
SD-OCT: spectral-domain optical coherence tomography
SE: spherical equivalence
SIM: superior-inner macular sectors
SOM: superior-outer macular sectors
TD-OCT: time-domain optical coherence tomography
TML: total macular layer
VF: visual field
